# Construction of a *Zygosaccharomyces rouxii* strain overexpressing the *QOR* gene for increased HDMF production

**DOI:** 10.1002/fsn3.4109

**Published:** 2024-03-18

**Authors:** Yanhong Wang, Xinhui Wang, Peng Jiang, Lingyan Dai, Yijia Hu, Bailing Pan, Yueyue Li, Jingyu Zhang, Ruoyu Zhang, Shihan Zhan, Zhijiang Li

**Affiliations:** ^1^ Heilongjiang Provincial Key Laboratory of Environmental Microbiology and Recycling of Argro‐Waste in Cold Region, Department of Bioengineering, College of Science and Biotechnology Heilongjiang Bayi Agricultural University Daqing Heilongjiang China; ^2^ Department of Food Science and Engineering, College of Food Science Heilongjiang Bayi Agricultural University Daqing Heilongjiang China; ^3^ Heilongjiang Engineering Research Center for Coarse Cereals Processing and Quality Safety Heilongjiang Bayi Agricultural University Daqing Heilongjiang China

**Keywords:** 4‐hydroxy‐2,5‐dimethyl‐3(2H)‐furanone, genetically engineered strain, *QOR* gene, quinone oxidoreductase, *Zygosaccharomyces rouxii*

## Abstract

4‐Hydroxy‐2,5‐dimethyl‐3(2H)‐furanone (HDMF) is a flavor compound widely found in natural products and is used in food as a flavor‐enhancing agent. Quinone oxidoreductase (QOR) was verified as a key enzyme to synthesize HDMF in strawberry, while its impact on HDMF production by *Zygosaccharomyces rouxii* was still unknown. The *QOR* gene was cloned and overexpressed in *Z. rouxii*, and its impact on HDMF production by *Z. rouxii* was then further analyzed. At the same time, it is expected to obtain engineered strains of *Z. rouxii* with high HDMF production. The results showed that the engineered strains of *Z. rouxii* exhibit different levels of *QOR* gene expression and HDMF production; among them, the QOR6 strain exhibiting the highest gene expression level and HDMF production was named as *ZrQOR*. The HDMF production of the *ZrQOR* strain was significantly higher than that of wild‐type *Z. rouxii* at 3 and 5 days of culture, with 1.41‐fold and 1.08‐fold increases, respectively. At 3 days of fermentation, the highest HDMF yield of *ZrQOR* strain was obtained (2.75 mg/L), 2 days ahead of the reported highest HDMF production by *Z. rouxii*. At 3, 5, and 7 days, *QOR* gene expression was 4.8‐fold, 3.3‐fold, and 5.6‐fold higher in the *ZrQOR* strain than in the wild‐type *Z. rouxii*, respectively. Therefore, overexpression of the *QOR* gene facilitates HDMF synthesis. The genetic stability of the 0–20 generation *ZrQOR* strain was stable, and there was no significant difference in colony shape, *QOR* expression, or HDMF production compared to the wild type. In this study, the genetic engineering *Z. rouxii* strain was used to improve HDMF production. This research has laid the groundwork for further industrial production of HDMF via microbial synthesis.

## Graphical abstract

This study proposed a formation pathway from exogenous nutrients to 4‐hydroxy‐2,5‐dimethyl‐3(2H)‐furanone (HDMF). During this procedure, quinone oxidoreductase (QOR) is a key enzyme for HDMF production. Therefore, the *QOR* gene was cloned and overexpressed in *Zygosaccharomyces rouxii*, and its impact on HDMF production by *Z. rouxii* was then further analyzed.

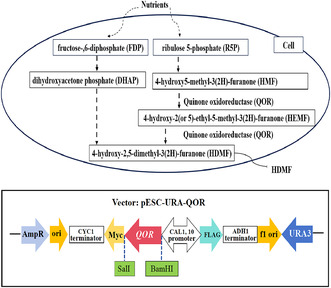

## 1 INTRODUCTION

*Zygosaccharomyces rouxii* is a salt‐tolerant yeast used in traditional fermentation products. During fermentation, *Z. rouxii* produces furanones, such as 4‐hydroxy‐2,5‐dimethyl‐3(2H)‐furanone (HDMF) and 4‐hydroxy‐2(or 5)‐ethyl‐5‐methyl‐3(2H)‐furanone (HEMF), which have a caramel odor. Therefore, it is an important aroma‐producing yeast with potential industrial value (Hayashida et al., 1999; Li, Zhao, et al., 2018; Li, Zhou, et al., 2018). HDMF is popular in several fields as a fragrance and aroma enhancer with a low odor threshold value of approximately 1–10 μg/mL (Buttery, 1999; Hauck et al., 2003). Accompanied by the improvement of people's living standards, the demand for HDMF in the food industry is increasing. However, the production of HDMF using *Z. rouxii* involves a high cost of the precursor substance fructose 1,6‐diphosphate (FDP) (Wang et al., 2009). Therefore, cost reduction and yield improvement are topics of intense interest in current research (Raab et al., 2006).

**FIGURE 1:**
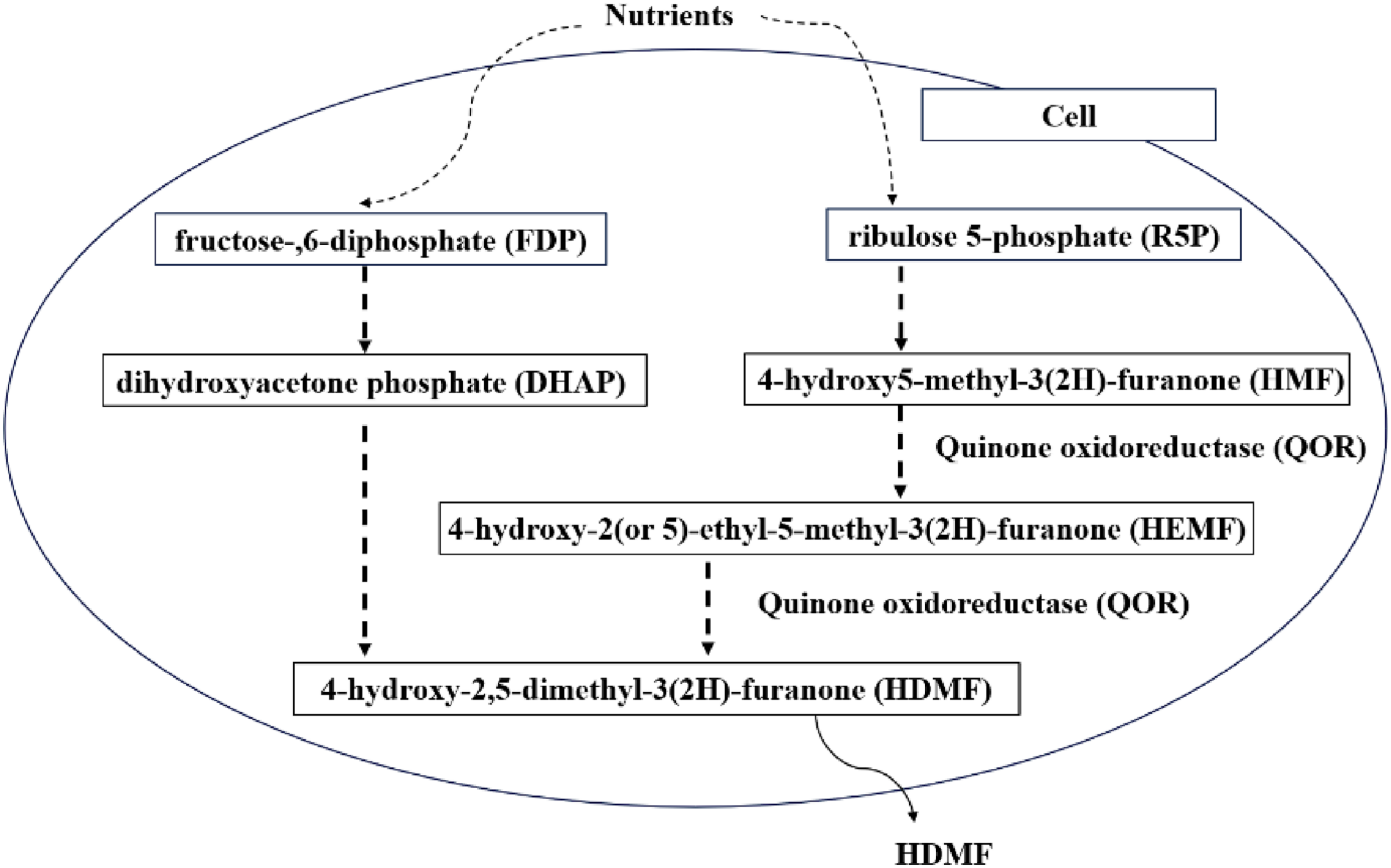
Proposed formation pathway from exogenous nutrients to HDMF. HDME, 4‐Hydroxy‐2,5‐dimethyl‐3(2H)‐furanone.

Quinone oxidoreductase (QOR) exhibits selectivity toward quinone substrates and serves as a crucial catalyst during the synthesis of HDMF through FDP metabolism in yeast (Hecquet et al., 1996; Landry et al., 2021; Zhang et al., 2018). QOR was discovered in the biosynthetic pathway of HDMF and its derivatives in strawberries (Yamada et al., 2018). QOR was found to be the last enzyme involved in the HDMF synthesis pathway and is functionally expressed in *Escherichia coli*; thus, it was hypothesized that QOR can catalyze HDMF production via 4‐hydroxy‐5‐methyl‐2‐methylene‐3(2H)‐furanone (HMMF) (Raab et al., 2006). Further studies revealed the role of QOR in HMMF for the synthesis of HDMF by hydrogenation of a previously unknown HMMF or 4‐hydroxy5‐methyl‐3(2H)‐furanone (HMF) derivatives substituted at the methylene functional group, rather than reduction of the double bond of straight‐chain 2‐alkenals or 2‐alkenones, and this pathway is operative in tomato fruit (Klein et al., 2007). The exogenous nutrients and their intermediate metabolites, such as those FDP involved in EMP pathway and ribulose 5‐phosphate (R5P) in PP pathway, were also verified as precursors (Li et al., 2020). Thus, QOR and other enzymes involved play important roles in regulating the synthesis of HDMF, as shown in Figure 1.

The construction of engineered yeast mainly occurs through the regulation of metabolic pathways and the construction of synthetic pathways for the target product in the host cell. The construction of genetically engineered yeast with the desired characteristics is efficient and environmentally friendly and might be produced in large quantities (Rahmat & Kang, 2020). To date, the use of *Saccharomyces cerevisiae* to build engineered strains with high production of hydroxytyrosol (Liu et al., 2022) and p‐coumaric acid (Zhang et al., 2020) and the use of *Pichia pastoris* to produce keratinase (Xu et al., 2019) have achieved good results. However, studies on the use of *Z. rouxii* to construct genetically engineered yeast for high yields of HDMF have not been reported. Some modern biotechnologies, such as genetic engineering technology, can be used to construct genetically engineered strains with high HDMF production. This can achieve the characteristics of economy, efficiency, and environmental friendliness in the large‐scale production of HDMF using microbial factories.

The aim of this study was to clone the *QOR* gene and overexpress it in *Z. rouxii*. The genetically engineered strain with higher production of HDMF was then screened through HDMF production and gene expression. Furthermore, the genetic stability and HDMF‐produced function of this engineering strain were analyzed, which will provide a theoretical basis for enterprises to further utilize engineering strains on a large scale to produce natural HDMF.

## 2 MATERIALS AND METHODS

### 2.1 Materials and main reagents

Native *Z. rouxii* (ATCC 2624) was obtained from the China General Microbiological Culture Collection Center. The overexpression vector was constructed by Wuhan Transduction Biological Laboratory (Wuhan, China). Yeast peptone dextrose medium (YPD, containing 20.0 g/L peptone, 20.0 g/L glucose, 10.0 g/L yeast extract, and 180 g/L NaCl) was obtained from Qingdao Hope Bio‐Technology Co., Ltd. (Qingdao, China). The standard for HDMF was obtained from Sigma Aldrich (St. Louis, MO, purity ≥97%).

### 2.2 Cultivation of *Z. rouxii*

According to the method of Fu et al. (2022), with slight modifications, the freeze‐dried powder of native *Z. rouxii* was activated. In a biological clean room, a sample was added to 100 mL of sterilized YPD broth and incubated for 3–4 days at 28°C and 180 rpm in a temperature‐controlled shaking incubator. The culture was stored when cell counts were measured with a hemacytometer at 2 × 10^8^ CFU/mL. The activated seed was inoculated at 5% (approximately 2 × 10^8^ CFU in 1 mL of solution) into a new 100 mL of sterile YPD and incubated at 28°C and 180 rpm for 30–35 h. The seeded fermentation broth was obtained when the total number of cells reached 2 × 10^8^ CFU/mL. Seed broths in triplicate were added to the sterilized medium at 5%, then each of them was incubated at 0, 3, 5, and 7 days, and then those were subsequently added with an equal volume of 70% glycerol into the strains, mixed well, and stored at −80°C in the fridge. Biomass calculation was carried out according to the method of Li et al. (2020).

### 2.3 PCR amplification and construction of the overexpression vector

The *QOR* gene sequence (AC: XM_002494472.1) from native *Z. rouxii* was first obtained from the NCBI database, and primers were designed using *Primer Premier* 5.0 Software (Premier Biosoft Co. Ltd., Palo Alto, California, USA) (Table 1). The genomic DNA was extracted using the yeast genomic DNA extraction kit (Qualityard Biotechnology Co., Ltd.), and the specific process was carried out according to its instructions. Using genomic DNA as a template, the *QOR* gene was then amplified by PCR using the 2 × PCR MiX enzyme (a high‐fidelity enzyme) and finally observed by 1% agarose gel electrophoresis. The PCR program was set to predenaturation at 95°C for 5 min, denaturation at 95°C for 30 s, annealing at 55°C for 30 s, and extension at 72°C for 1 min for a total of 35 cycles. The *ZrQOR* gene was purified by the gel recovery method, connected to the vector pESC‐MCS2 with a strong GAL promoter and Amp + resistance, and inserted between the polyclonal sites 5′ BamHI‐SalI 3′. The ligated product was transferred into *E. coli* DH5 alpha, the plasmid was extracted, and the construction of the target gene recombinant, named pESC‐URA‐QOR, was identified by 1% agarose gel electrophoresis.

**TABLE 1:** Primers sequence design.

Primer name	Sequences (5′ to 3′)	Product length (bp)
QOR‐QRT‐F	ACCACAACTATTCCAAAGACCCA	200
QOR‐QRT‐R	CCTAGGATCAGTGGCTTTTCACT	
QOR‐F	CGCGGATCCGATGCTTAGATCGACAATAT	1066
QOR‐R	GACGTCGACTTGAGGAATCTCCAAGACT	
QOR‐GAL‐F	GTTGTGGAAATGTAAAGAGCCCC	632
QOR‐GAL‐R	TGGGTCTTTGGAATAGTTGTGGT	
QOR‐CYC‐F	AGGTTGATGCTGCTGGTAAAGTA	931
QOR‐CYC‐R	ACCTTCTCAAGCAAGGTTTTCAG	
AMP‐F	CGTCGTTTGGTATGGCTTCATTC	581
AMP‐R	TCCGCTCATGAGACAATAACCCT	
GAPDH‐F	AGACTGTTGACGGTCCATCC	121
GAPDH‐R	CCTTAGCAGCACCGGTAGAG	

*Note*: Underlines in primer sequences mean the cleavage site of the enzyme.

### 2.4 Acquisition of the positive transformants

First, the recombinant plasmids in *E. coli* were extracted using an OMEGA Plasmid Mini Kit I. Overexpression of the *QOR* gene in a yeast strain was constructed according to the method of Wang et al. (2022) with minor modifications. A total of 1–5 μg of purified plasmid and 100 μL of receptor cells were mixed, transferred to an electrotransfer cup, and electroshocked for 5 milliseconds at 2.1 kV. After shocking, 1 mL of YPD was quickly added to the electrotransfer cup, and the cells were gently suspended before being transferred to a 1.5 mL centrifuge tube and resuscitated for 2 h at 28°C and 180 rpm. Finally, the cell was cultured for 2–3 days in solid YPD medium until a single colony was formed. The positive clones were screened through colony PCR using multiple pairs of primers. The positive transformants with *QOR* gene overexpression were subsequently referred to the engineered yeast strain.

### 2.5 RNA extraction, cDNA synthesis, and qRT‐PCR analysis

Total RNA from native *Z. rouxii* and engineered yeast strain samples was obtained using TRIzol reagent (Invitrogen, Carlsbad, CA, USA). The quality of the extracted RNA was determined using agarose gel electrophoresis. A Nanodrop 2000 (Thermo Scientific, Waltham, MA, USA) was used to determine the concentration of extracted RNA samples. Then, ReverTra Ace qPCR RT Master Mix with a gDNA Remover kit (TOYOBO, Dalian, China) was used for cDNA synthesis. The diluted cDNA was taken, and qRT–PCR was performed using THUNDERBIRD SYBR® qPCR Mix (TOYOBO). The qRT–PCR program was set to predenaturation at 95°C for 5 min, denaturation at 95°C for 30 s, annealing at 60°C for 30 s, and extension at 72°C for 30 s, for a total of 30 cycles, and NCBI/Primer‐BLAST was used to design the primers. The gene expression levels were measured after 0, 3, 5, and 7 days of fermentation, and the gene expression in the original group was set as the control group. After being fermented for 0, 3, 5, and 7 days, the *QOR* gene expression levels were measured in *Z. rouxii* and the transformed yeast, and the *GAPDH* gene was used as an internal reference gene. Three biological and technical replicates were measured, and the relative gene expression was calculated using the 2^−∆Ct^ method (Elcik et al., 2021).

### 2.6 Determination of the HDMF content

**FIGURE 2:**
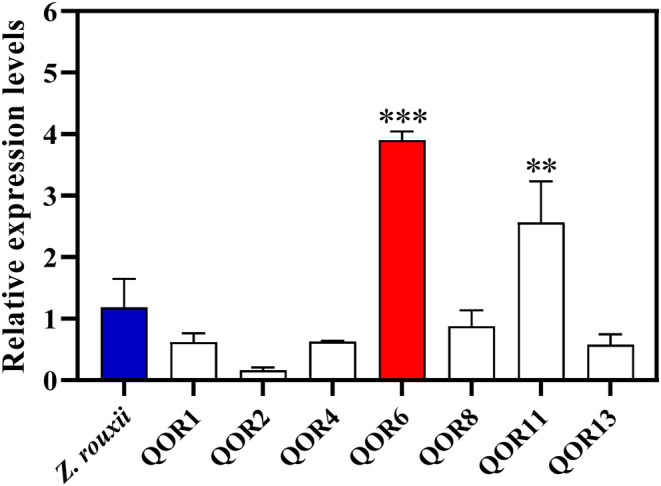
*QOR* gene expression of positive clones and native *Zygosaccharomyces rouxii*, based on the qRT–PCR results. *Representing *p* < .05, **representing *p* < .01, and ***representing *p* < .001 are considered as statistically significant, respectively, the same as below. QOR, quinone oxidoreductase.

According to the modified method (Li et al., 2020), the HDMF content was determined on a High Performance Liquid Chromatography (HPLC) system equipped with an Agilent 1260 Infinity II UV–VIS Diode Array Detector (DAD, Agilent Technologies, CA, USA). The fermentation broth was centrifuged at 8000 rpm for 10 min, and the supernatant was transferred to a freshly sterilized centrifuge tube and filtered through a 0.22 μm membrane before loading. The column was a ZORBAX Eclipse XDB‐C18 column (5 μm, 250 × 4.6 mm, Agilent) with a mobile phase of 0.5% formic acid and an injection volume of 10 μL. A gradient starting at 95% A (0.5% formic acid in water) and 5% B (acetonitrile) to 80% A within 10 min and then to 0% A in 15 min was used at a flow rate of 1 mL/min and recorded at a wavelength of 287 nm. HDMF standard chromatography is shown in Figure 1S. The linearity of a standard curve using an external standard was assessed over the range of 5–90.0 mg/mL for HDMF. The linear regression equation for HDMF is *y* = 32.397× + 3.904 (Figure 2S, *R* ^2^ > 0.9997, 98.5%–103.5% accuracy).

### 2.7 Genetic stability analysis

The *ZrQOR* strains were inoculated on YPD plates and incubated at 28°C for 3 days to check the morphology of the colonies. The cell morphology was also examined using an electron microscope (Chongqing Optics & Electronics Instrument Co., Ltd., China). The HDMF production and *QOR* gene expression in engineered yeast at 0, 5, 10, 15, and 20 generations were determined by referring to the above method at 3 days; each 10–15 h was regarded as one generation.

### 2.8 Statistical analysis

All tests were performed in triplicate, and expressed as the mean ± SD deviation. The data were analyzed using *SPSS* 20.0 (SPSS, Inc., Chicago, IL, USA), and an independent sample *t* test was used to compare the data for the two groups. *p* < .05, *p* < .01, or *p* < .001 indicates that the data were statistically significant. The figures were prepared with *GraphPad Prism* 8.0 (GraphPad Software, Inc., San Diego, CA, USA).

## 3 RESULTS AND DISCUSSION

### 3.1 Determination of *QOR* gene expression

**FIGURE 3:**
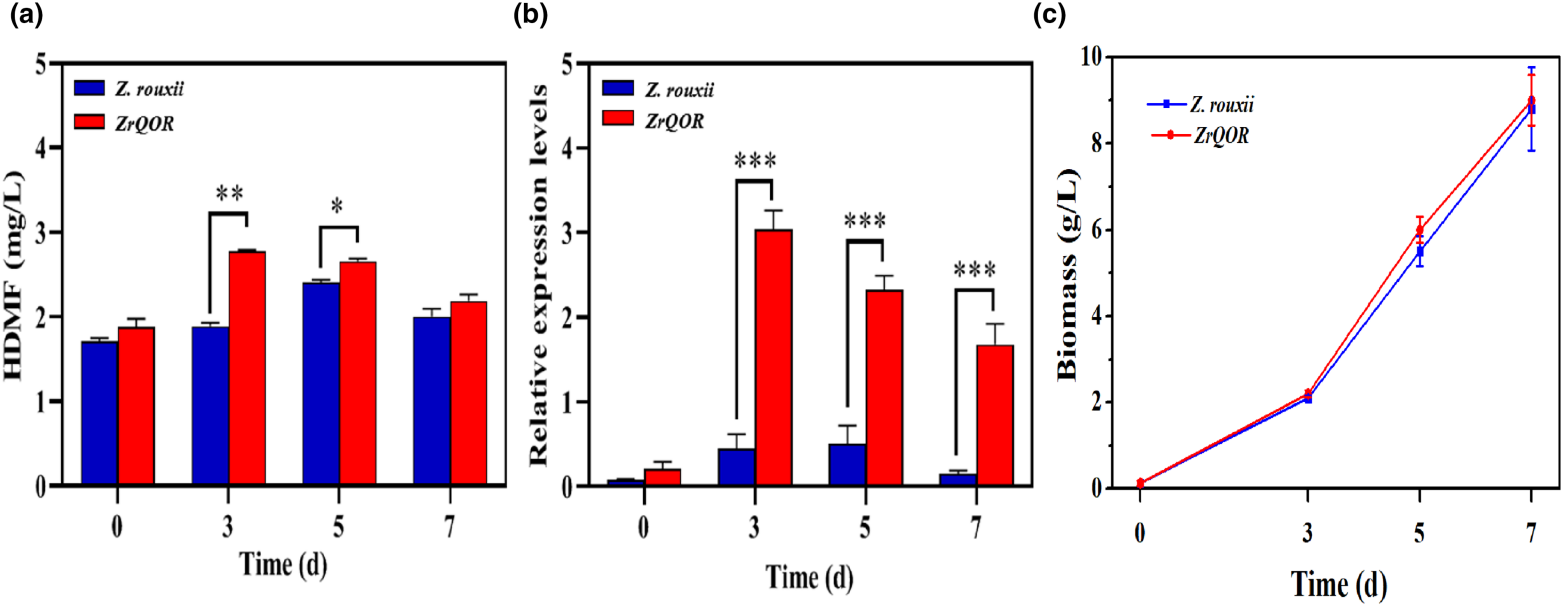
HDMF production (a), relative expression levels of *QOR* gene (b), and biomass (c) at various periods in QOR6. HDME, 4‐Hydroxy‐2,5‐dimethyl‐3(2H)‐furanone; QOR, quinone oxidoreductase.

**FIGURE 4:**
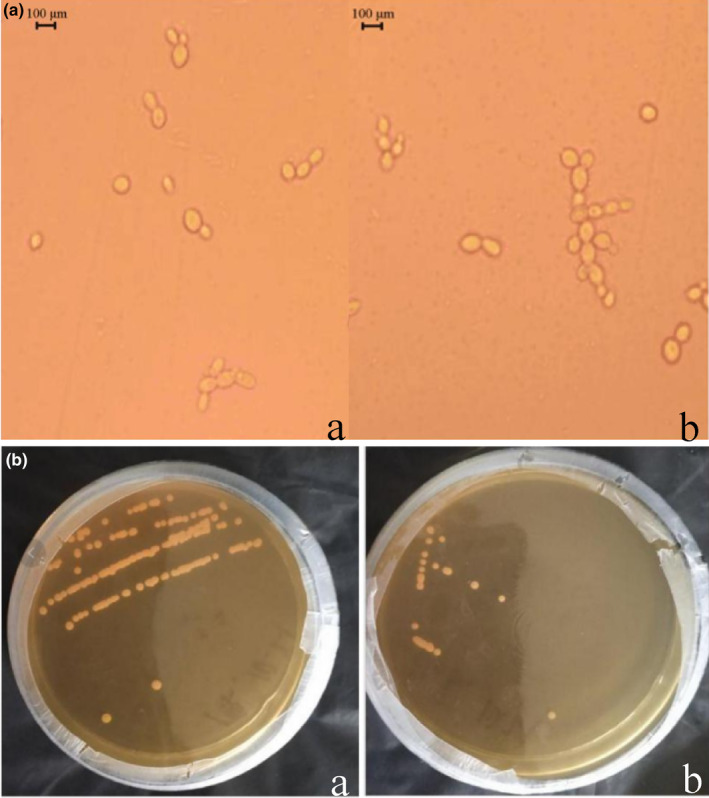
Observation of *ZrQOR* strain cell morphology (a) and colony morphology (b) in QOR6. a, original generation; b, 20th generation. HDME, 4‐Hydroxy‐2,5‐dimethyl‐3(2H)‐furanone; QOR, quinone oxidoreductase.

Cloning and overexpression vector construction of the *QOR* gene (Supplementary Material 1—Data S1, Figure 3S) and screening of 7 positive clones of the *ZrQOR* strain (Supplementary Material 2—Data S1, Figure 4S) were prepared. To confirm whether the *QOR* gene was overexpressed in the 7 positive clones, *QOR* gene expression was measured by qRT–PCR at 3 days of fermentation using *GAPDH* as the internal reference gene. According to the results in Figure 2, the *QOR* gene's relative expression of QOR6 and QOR11 clones was significantly higher than those of the native *Z. rouxii*, with 3.2‐fold (*p* < .001) and 2.1‐fold (*p* < .01) higher expression, respectively. Therefore, QOR6 and QOR11 were identified as effective engineered yeasts overexpressing QOR. QOR6 showed the highest expression, so it was used in the subsequent experiments.

### 3.2 HDMF production and relative expression of QOR6 in different culture periods

The HDMF content was measured at different fermentation times using HPLC. The results are shown in Figure 3a. At 3 and 5 days, QOR6 produced significantly more HDMF than native *Z. rouxii* (1.41‐fold and 1.08‐fold increases, respectively) (*p <* .01 & *p* < .05). At 5 days of fermentation, the highest HDMF yield of native *Z. rouxii* was 2.35 mg/L, while the highest HDMF yield of QOR6 was 2.75 mg/L at 3 days of fermentation. Therefore, almost 2 days of fermentation time reduction were conducted at the level of similar HDMF production between native *Z. rouxii* and genetic strains. Regardless of the presence or absence of D‐fructose in YPD culture medium, the highest HDMF production was observed in *Z. rouxii* on the fifth day of fermentation (Li et al., 2020; Zhou, 2018). It is evident that overexpression of the *QOR* gene in *Z. rouxii* leads to an earlier peak in HDMF synthesis by 2 days. HDMF production by QOR6 initially increased and then decreased, which was the same as the trend of HDMF production by native *Z. rouxii*. It is possible that HDMF production decreased beginning at 7 days due to substrate depletion in the medium, which is consistent with previously reported results (Li et al., 2020; Li, Zhao, et al., 2018; Li, Zhou, et al., 2018). However, compared with the addition of FDP, the amount of HDMF produced by the *ZrQOR* strain was lower (Li et al., 2020). *Pichia guilliermondii* HDMF production was up to 92.5 mg/L in 100 g/L FDP medium (Zhang, 2009). After 5 days of complete fermentation in YPD supplemented with 120 g/L D‐fructose and 180 g/L NaCl, HDMF production by native *Z. rouxii* reached 6.77 mg/L (Zhou, 2018). This result indicates that external supplementation with a carbon source is essential for HDMF production in native *Z. rouxii* (Thomas et al., 2001).

For dynamic analysis of QOR expression in engineered yeast (Figure 3b), QOR6 and native *Z. rouxii* were inoculated into YPD for simultaneous cultivation. Expression in both yeasts at 0, 3, 5, and 7 days was determined by qRT‐PCR. Expression in QOR6 was found to be significantly higher than that in native *Z. rouxii* at 3, 5, and 7 days of fermentation (*p* < .001), with 4.8‐fold, 3.3‐fold, and 5.6‐fold higher expression, respectively. The overall trend in relative *QOR* gene expression followed the same trend as HDMF production, first increasing and then decreasing. And biomass between the QOR6 and *ZrQOR* strains indicated a similar tendency (Figure 3c). Based on the above results, *QOR* gene expression and HDMF yield were positively correlated. This finding indicates that QOR, as a key catalyst for the biosynthesis of HDMF in *Z*. *rouxii*, can promote the production and accumulation of HDMF via the genetically engineered yeast, thus increasing the yield of HDMF.

### 3.3 Observation of colony morphology and cell morphology

Due to the existence of repair mechanisms in yeast strains, not all transformed strains are able to stably maintain their beneficial traits (Hülter et al., 2020). Therefore, genetic stability analysis of strains is needed.

The cell morphology of each generation was observed by electron microscopy, as shown in Figure 4a. Generations 1–20 were all oval‐shaped, and there was no significant difference in the morphology compared to the original generation of the engineered yeast. The morphology of the colonies of the original and 20th generations of the *ZrQOR* strain is shown in Figure 4b, with a white, round, and smooth appearance, typical of yeast colonies. There was no significant difference between the generations, the morphology was the same as that of the original generation, and there were no aberrant colonies.

### 3.4 Detection of target fragments

During successive iterations of engineered strains, plasmid replication is often delayed compared to cell division, resulting in the loss of the target plasmids in the engineered strain (Zheng et al., 2021). Therefore, it is crucial to verify the stability of the plasmids carried by recombinant engineered strains to cultivate high‐quality recombinant engineered strains. In this experiment, PCR was performed to verify the presence of plasmids in the 1st, 5th, 10th, 15th, and 20th generations of QOR6. This finding shows that the genotype of the *ZrQOR* strain remained unchanged after 20 generations and has high genetic stability.

### 3.5 Analysis of HDMF production and stability of *QOR* gene expression by generations of QOR6

**FIGURE 5:**
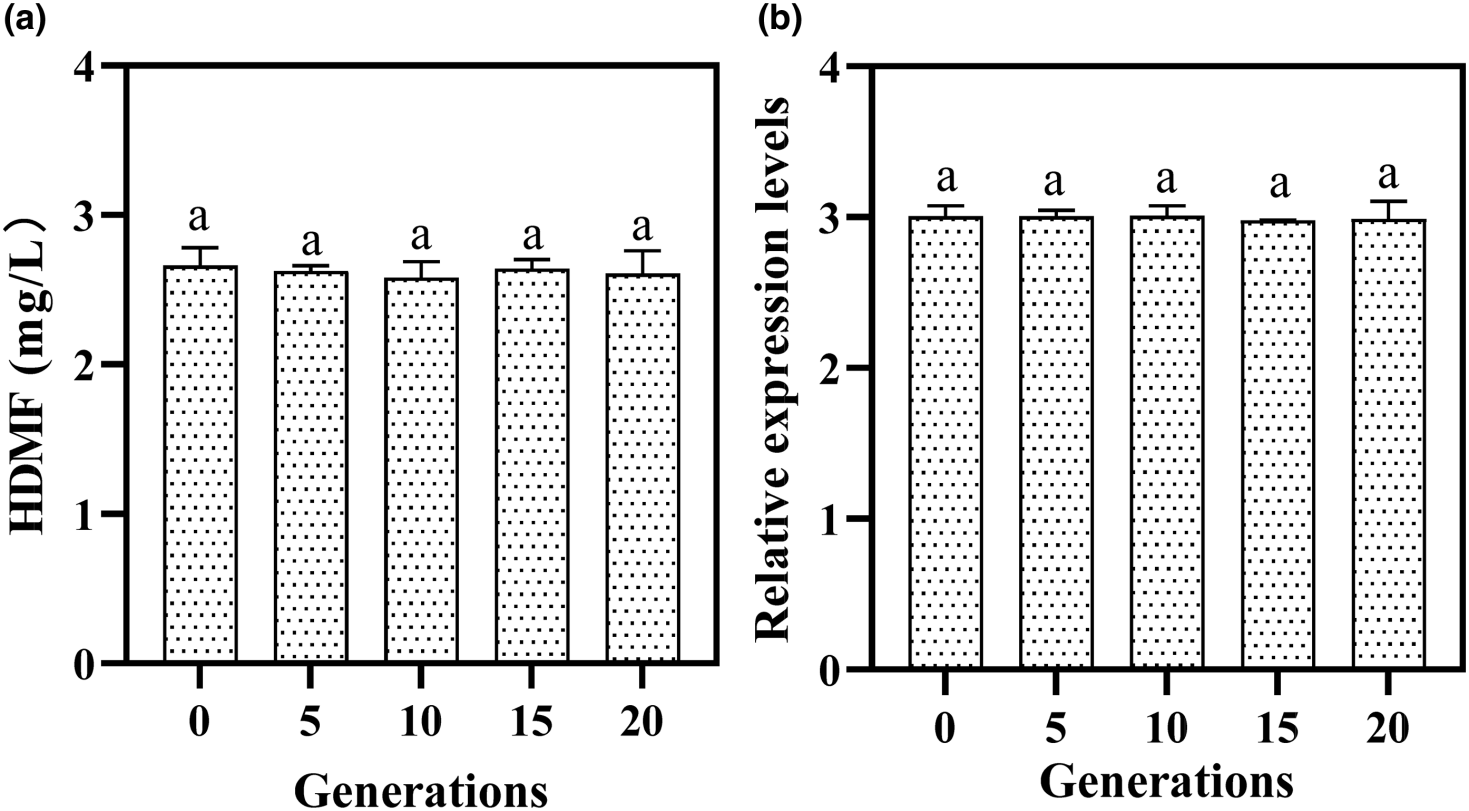
*ZrQOR* genetic stability analysis and HDMF production of QOR6 at 1–20th generations. a, no significant difference between generations (*p* > .05). (a) is the HDMF yield by *ZrQOR*, and (b) is the relative expression of *QOR* gene. HDME, 4‐Hydroxy‐2,5‐dimethyl‐3(2H)‐furanone; QOR, quinone oxidoreductase.

The production of HDMF is directly correlated with the growth of *Z. rouxii* (Thomas et al., 2001). The HDMF yield was measured after 3 days of incubation for each QOR6 generation from 0, 5, 10, 15, and 20. As shown in Figure 5a, the yield of HDMF was relatively stable at approximately 2.6 mg/L among all generations of QOR6. There was no significant difference in yield among generations, indicating that the HDMF yield was relatively stable at the 20th generation of inheritance. Therefore, the *QOR* gene can be stably inherited, and the yield of HDMF by the *ZrQOR* strain is significantly higher than that of *P. pastoris* (4 days and 2 mg/L) (René et al., 1997) and *Lactobacillus* (3 days and 1.17 mg/L) (Hayashida et al., 2001). The relative expression of the *QOR* gene was measured after culturing each generation of QOR6 (0, 5, 10, 15, and 20) for 3 days. As shown in Figure 5b, the expression of the *QOR* gene in generations 0, 5, 10, 15, and 20 of QOR6 was approximately 3, and there was no significant difference in expression (*p* > .05). This result indicates that *QOR* gene expression was relatively stable in the 20th generation.

## 4 CONCLUSION

Although FDP is the most favorable precursor substance found to promote HDMF production, it is not an ideal precursor substance for industrial production because of its high cost. Therefore, it is a new trend in development to combine fermentation engineering with genetic engineering to construct engineered yeast for producing target products. In this study, we successfully transferred the gene for the key enzyme involved in HDMF synthesis into native *Z. rouxii*. A genetically stable *ZrQOR* strain was developed with a brief cultivation period, producing a high HDMF yield. The objectives of time reduction and yield enhancement were successfully attained. However, the yield of HDMF was still lower in comparison with an exogenously added carbon source. Therefore, finding a suitable carbon source is necessary to increase the yield of HDMF. It is therefore necessary to continue studies on improving HDMF yield in the *ZrQOR* strain itself and its metabolic mechanism. Further pilot scale production is possible to produce HDMF in large quantities by the microbiological method in factories.

## AUTHOR CONTRIBUTIONS

**Yanhong Wang:** Conceptualization (equal); data curation (equal); investigation (equal); methodology (equal); software (equal); writing – original draft (equal); writing – review and editing (equal). **Xinhui Wang:** Writing – review and editing (equal). **Peng Jiang:** Writing – review and editing (equal). **Lingyan Dai:** Conceptualization (equal); data curation (equal); investigation (equal); methodology (equal); writing – original draft (equal); writing – review and editing (equal). **Yijia Hu:** Conceptualization (equal); data curation (equal); investigation (equal); methodology (equal); writing – original draft (equal). **Bailing Pan:** Conceptualization (equal); data curation (equal); investigation (equal); methodology (equal); software (equal); writing – original draft (equal). **Yueyue Li:** Methodology (equal); software (equal); writing – review and editing (equal). **Jingyu Zhang:** Data curation (equal); software (equal); writing – review and editing (equal). **Ruoyu Zhang:** Data curation (equal); writing – review and editing (equal). **Shihan Zhan:** Investigation (equal); writing – review and editing (equal). **Zhijiang Li:** Conceptualization (lead); funding acquisition (supporting); supervision (lead); writing – review and editing (lead).

## FUNDING INFORMATION

This work was supported by the Natural Science Foundation of Heilongjiang Province of China (LH2022C059), the Postdoctoral Scientific Research Developmental Fund of Heilongjiang Province (LBH‐Q20056 & LBH‐Q19164), the Heilongjiang Bayi Agricultural University Support Program for San Heng San Zong (ZRCPY202028 & ZRCLG201906), the Open Competition Mechanism to Select the Best Key Science and Technology Program of Heilongjiang Bayi Agricultural University (JB20220001), and College Students Innovative Entrepreneurial Training Plan Program in Heilongjiang (S202310223102X).

## CONFLICT OF INTEREST STATEMENT

The authors declare that the research was conducted in the absence of any commercial or financial relationships that could be construed as a potential conflict of interest.

## PUBLISHER'S NOTE

All claims expressed in this article are solely those of the authors and do not necessarily represent those of their affiliated organizations, or those of the publisher, the editors, and the reviewers. Any product that may be evaluated in this article, or any claim that may be made by its manufacturer, is not guaranteed or endorsed by the publisher.

## Supporting information


Data S1:

## Data Availability

The data underlying this article will be shared on reasonable request to the corresponding author.
