# Supplementary material for: Construction of a Zygosaccharomyces rouxii strain overexpressing the QOR gene for increased HDMF production
Source: Food Sci Nutr. 2024 Mar 18;12(6):4435–42. doi: 10.1002/fsn3.4109 (PMC11167138; doi:10.1002/fsn3.4109)
Supplement: Supplementary file 1 — Data S1: [file FSN3-12-4435-s001.docx]

**Supplementary files**

**Supplementary material 1:**

**Cloning and overexpression vector construction of *QOR* gene**

*QOR* gene with a total length of 1050 bp in *Z. rouxii* was amplified using PCR technology (Fig. 3S, A), located on chromosome A and encoding 349 amino acids. Compared QOR proteins between *Z. rouxii* and strawberry (*Fragaria × ananassa*), alignment was only 25.71%, indicating that difference was significant. The dot-matrix plot of protein sequence alignment analysis shown that these two proteins indicated higher similarities in partial distribution which was presented through multiple separated small fragments (Fig. 3S, B). As shown in Fig. 3S, C, the empty vector used was pESC-MCS2, a high copy yeast expression vector, and constructed by the Wuhan Transduction and Refinement Biotechnology Co., Ltd. The *QOR* fragment from native *Z. rouxii* was inserted into the polyclonal site 5' BamHI-SalI 3' to obtain the *QOR* gene overexpression vector, which was 7651 bp in length.

(A): QOR gene cloning; M, Marker DL2000; C, negative control; S, QOR gene from *Z. rouxii*. (B): Dot-matrix plot of QOR protein sequence alignment between *Z. rouxii* and *Fragaria × ananassa.* The numerical values on the horizontal and vertical axes represent the position of amino acids. (C): pESC-URA-QOR vector.

**Figure 3S** Cloning of *QOR* gene, protein alignment analysis, and construction of gene overexpression vector.

**Supplementary material 2:**

**Screening of positive clones of *ZrQOR* strain**

*Z. rouxii* is the main yeast that produces HDMF, an aromatic substance. However, unlike *Pichia pastoris*, there are no defective strains of *Z. rouxii* available for theoretical research. Therefore, the positive clones in this study were validated through colony PCR using multiple pairs of primers. We designed three primers, QOR-GAL, QOR-CYC, and AMP, at the promoter, terminator, and resistance fragment of the constructed vector, respectively. Firstly, primer AMP was used for the first round of positive clones screening (Supplementary Fig. 4S, A). Subsequently, primer QOR-GAL was used to screen for the previously identified positive clones (Supplementary Fig. 4S, B). Finally, primer QOR-CYC was used to further screen for the positive clones (Supplementary Fig. 4S, C). The extracted plasmid was used as a positive control, and sterilized water was used as a negative control to screen positive colonies. The three screenings showed expected electrophoretic bands from colonies 1, 2, 4, 6, 8, 11, and 13 (Supplementary Fig. 4S, C). There was no band in the negative control of the C lane, indicating that the samples were free of contamination, and the positive control of the Z lane had a single band. Therefore, the 7 colonies in Supplementary Fig. 4S, C were identified as positive colonies, named QOR1, QOR2, QOR4, QOR6, QOR8, QOR11, and QOR13.


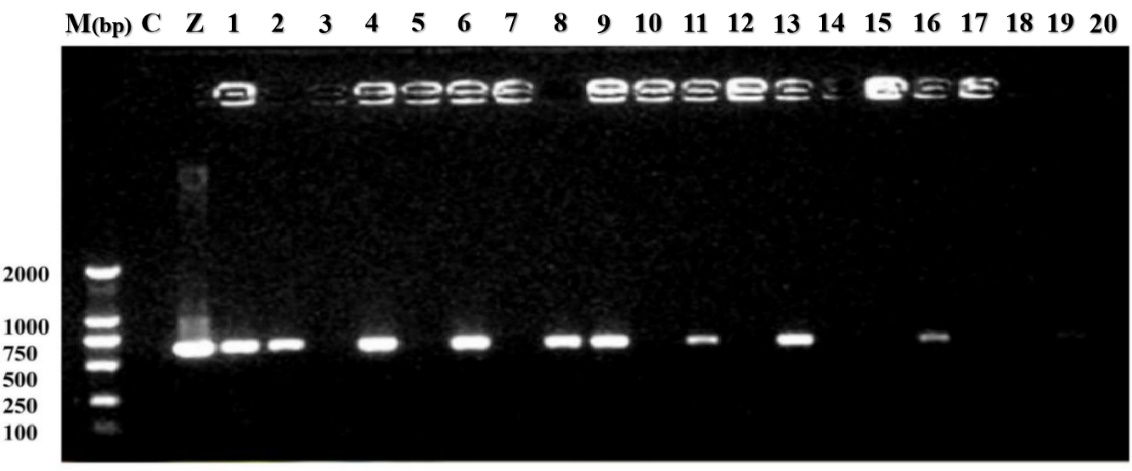


**A**

M, Marker DL2000; C, negative control; Z, positive control; 1-20, 20 engineered colonies screened.


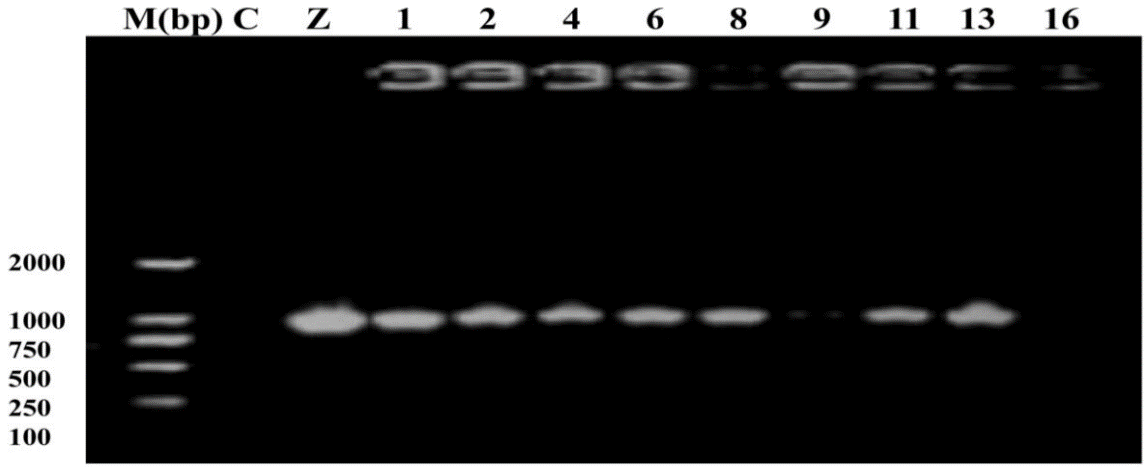


**B**

M, Marker DL2000; C, negative control; Z, positive control; 1-16, 9 engineered colonies screened.


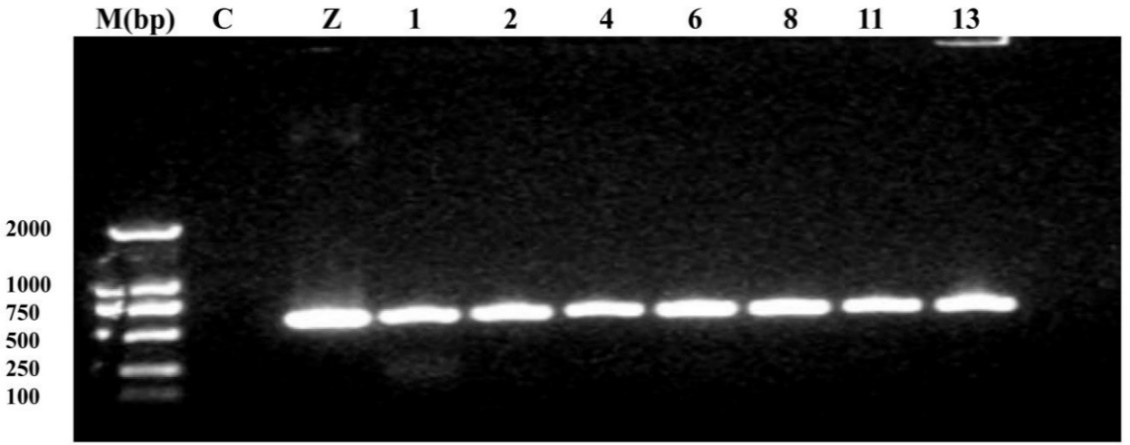


**C**

M, Marker DL2000; C, negative control; Z, positive control; 1-13, 7 engineered colonies screened.

**Figure 4S** PCR electrophoresis for positive colony screenings of *ZrQOR* strain using QOR-GAL (A), QOR-CYC (B) and AMP (C) as primers.

**Supplementary Figures:**


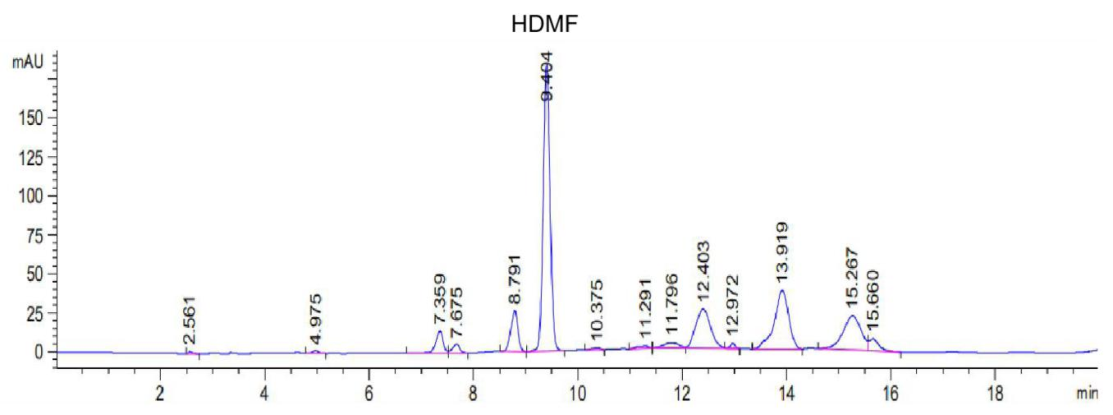


**Figure 1S** HDMF standard chromatography by HPLC.


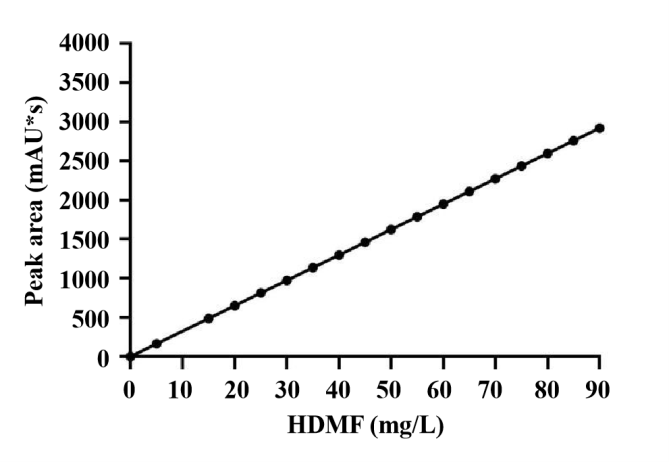


**Figure 2S** Standard curve of HDMF.
